# Mycobacteria as Environmental Portent in Chesapeake Bay Fish Species

**DOI:** 10.3201/eid1302.060558

**Published:** 2007-02

**Authors:** Andrew S. Kane, Cynthia B. Stine, Laura Hungerford, Mark Matsche, Cindy Driscoll, Ana M. Baya

**Affiliations:** * University of Maryland, Baltimore, Maryland, USA; † Virginia-Maryland Regional College of Veterinary Medicine, College Park, Maryland, USA; ‡ University System of Maryland, College Park, Maryland, USA; § Maryland Department of Natural Resources, Oxford, Maryland, USA; ¶ Maryland Department of Agriculture, College Park, Maryland, USA

**Keywords:** Mycobacteria, syndromic surveillance, fish, Atlantic menhaden, Chesapeake Bay, dispatch

## Abstract

Infection with environmental mycobacteria is increasing among many Chesapeake Bay fish species. Prevalence in juvenile Atlantic menhaden differed between tributaries and ranged from 2% to 57%. Mycobacterial infection may be a syndromic sentinel of altered environmental conditions that threaten aquatic animal health.

An ongoing epizootic of mycobacteriosis has been reported among striped bass, *Morone saxatilis* (*1*), in the Chesapeake Bay, 1of the largest and most productive estuaries in North America. Sampling and culture of striped bass from locations across the bay have led to the isolation of a number of distinct species of mycobacteria that occur alone or as polyinfections within individual fish (*2*–*4*). One contemporary approach to investigating such emerging infections is to use molecular techniques to focus on genetic characteristics and relatedness linking isolates (*5*). Alternatively, as with the opportunistic infections associated with human HIV, the key may not be solely the identity of the infecting bacteria. Rather, these pathogens may be a portent of more fundamental health disturbances that threaten multiple species within the Chesapeake Bay system. Laboratory studies suggest that other bay fish, such as Atlantic menhaden, are susceptible to multiple species of mycobacteria (*6*) similar to the variety of types isolated from infections in wild striped bass (*2*–*4*).

The purpose of our study was to survey a wider set of fish species, at multiple discrete locations within the Chesapeake Bay and its tributaries, for mycobacterial infection. Particularly, we examined juvenile Atlantic menhaden because they are a “keystone” species in the bay. Ecologically, Atlantic menhaden represent the highest level (taxonomically) filter feeder in the bay. This may be of notable consequence because menhaden filter enormous volumes of sediment and plankton to derive nutrition (*7*), and aquatic mycobacteria can have an affinity for growing on particles, biofilms and sediments, and to be incorporated into amoebae, algae, and other microorganisms (*8*,*9*). Furthermore, menhaden provide a critical forage base for other animals and support “recruitment” to the bay’s adult fisheries.

## The Study

Fish were collected by beach seine, cast net, or bank trap from the Choptank, Chicamacomico, Nanticoke, and Pocomoke Rivers of the Chesapeake Bay. Live fish were transported in oxygenated, insulated coolers to the University of Maryland Aquatic Pathobiology Center for examination and microbiology. Liver (from Atlantic menhaden) and spleen (other fish species) tissues were sampled aseptically, homogenized in Butterfield’s phosphate-buffered saline and plated on Middlebrook 7H10 agar (Difco, Detroit, MI, USA) supplemented with Bacto Middlebrook oleic acid, albumin, dextrose, catalase (Difco). Plates were assessed for colony growth after 2–8 weeks, and mycobacteria were identified to the genus level on the basis of colony morphology, growth characteristics, and gas chromatographic fatty acid methyl ester analyses. Prevalence of mycobacterial infection (proportion of sampled fish with positive culture results in each subgroup) was calculated for each species. Prevalence in juvenile Atlantic menhaden was compared among the river systems by using Fisher’s exact test.

Mycobacteria were recovered from Atlantic menhaden, white perch, blueback herring, largemouth bass, mummichog, striped killifish, winter flounder, weakfish, and spot (Table 1). No externally visible lesions were present on fish of any species sampled, except Atlantic menhaden. A low percentage (<10%) of the sampled Atlantic menhaden had visible signs of disease, mainly external, often perianal ulcers penetrating through the skin and the underlying musculature. Histologic results from a subsample of these fish indicated that these lesions were consistent with ulcerative mycosis.

**Table 1:** Prevalence of culture-confirmed mycobacteriosis among fish sampled from mid–Chesapeake Bay tributaries

Species (n)	% Culture-positive	95% Confidence interval
Atlantic menhaden (287) *Brevoortica tyrannus*	18	13.5–22.7
Blueback herring (17) *Alosa aestivalis*	12	1.5–36.4
Winter flounder (26) *Pleuronectes americanus*	12	2.5–30.2
Striped killifish (1) *Fundulus majalis*	100	2.5–100.0
Mummichog (3) *F. heteroclitus*	33	0.8–90.6
Largemouth bass (1) *Micropterus salmoides*	100	2.5–100.0
Weakfish (2) *Cynoscion regalis*	50	1.3–98.7
Spot (27) *Leiostomus xanthurus*	7	0.9–24.3
White perch (87) *Morone americana*	20	11.8–29.4

Prevalence of mycobacterial infection among wild-caught, juvenile Atlantic menhaden ranged from 2% to 57% by river system (Table 2). Atlantic menhaden from the Chicamacomico River had a notably higher prevalence of infection (p<0.001) than menhaden sampled from the other river systems.

**Table 2:** Prevalence of culture-confirmed mycobacteriosis among Atlantic menhaden from 4 Chesapeake Bay tributaries

River (n)	% Culture-positive*	95% Confidence interval
Nanticoke (60)	2^a^	0.04–8.9
Choptank (134)	10^a,b^	5.3–16.0
Pocomoke (46)	21^b^	11.0–36.4
Chicamacomico (47)	57^c^	42.2–71.7

*Small letters indicate significant difference from other river systems at p<0.01 (Bonferroni-corrected Fisher exact test probabilities).

## Conclusions

Mycobacteriosis in the Chesapeake Bay is a problem of much wider scope than the previously recognized epizootic in striped bass (*1*). In this report, we document infection in multiple fish species representing a range of life histories, water strata, and locations. Why mycobacteriosis has emerged in this setting is unclear: the new findings reshape future investigations from a single host–single pathogen focus to consideration of the ecology of multiple hosts and related pathogens within a dynamic system. Certain water quality criteria, including those associated with degraded habitats, lower pH, and higher organic content, have been reported to foster the growth of environmental mycobacteria (*9*). Other factors, such as increases in suspended particulates, biofilms, and even water dynamics associated with global warming, may support enhanced growth of environmental pathogens including mycobacteria (*9*,*10*). Metal and organic contamination, algal blooms, and low dissolved oxygen levels serve as environmental stressors in the Chesapeake Bay (*11*). Water quality has strong spatial heterogeneity and temporal flux, and these conditions could exacerbate both bacterial proliferation and host susceptibility.

Variability in prevalence across species and locations provides opportunities to determine the underlying ecology of emerging infections. Preliminary water quality data from 3 of the 4 Atlantic menhaden sampling locations at the time of fish collection show that pH was lower in the Chicamacomico (6.9, standard deviation [SD] ± 0.3) and Pocomoke Rivers (7.1, SD ± 0.3), where mycobacterial prevalence was higher, than in the Choptank River (8.3, SD ± 0.4), where prevalence was lower. Further, dissolved organic carbon was notably higher in the Chicamacomico (16.5 mg/L, SD ± 1.1) and Pocomoke Rivers (11.7 mg/L, SD ± 0.5) than in the Choptank River (4.4, SD ± 0.4).

The prevalence of infection in Atlantic menhaden is notable and may indicate the potential of this fish to amplify spread to other species. This fish is an essential link in the food chain.. The Atlantic menhaden fishery, the largest commercial fishery in the Chesapeake Bay, provides an important source of protein (as fish meal) in animal feeds for both agricultural and domestic pets, as well as oils rich in omega-3 fatty acids used in human and veterinary diet supplements and as bases for cosmetics.

Aquatic mycobacteria cause opportunistic infections and disease in humans, most commonly among those who are immunocompromised or have other serious diseases, or following a skin abrasion or penetrating wound (*12*,*13*). The prevalence of mycobacteriosis in this study raises concern for potential increases in human infections in the Chesapeake Bay region through contact with fish, as well as through recreational contact and drinking water (*8*,*9*,*13*–*15*).

Mycobacterial infections in bay fish may serve as a syndromic sentinel of an environmental state that already affects the health of multiple inhabitants of the region. Data from this and ongoing studies refocus attention on the complex, anthropogenically accelerated changes that may be altering the distribution of emerging diseases worldwide. The Chesapeake Bay is an important ecosystem with a large urban component, so understanding the epidemiology of this multispecies epizootic may improve not only the health of local inhabitants but also the prediction of other emerging infections.
